# A spatial analysis of the expanding roles of nurses in general practice

**DOI:** 10.1186/1472-6955-11-13

**Published:** 2012-08-07

**Authors:** Christopher Pearce, Sally Hall, Christine Phillips, Kathryn Dwan, Rachael Yates, Bonnie Sibbald

**Affiliations:** 1 Director of Research, Inner Eastern Melbourne Medicare Local and Adjunct Associate Professor, Monash University, Melbourne, Australia; 2 Academic Unit of General Practice and Community Health, Australian National University Medical School, Canberra, ACT, Australia; 3 Australian Primary Health Care Research Institute, Australian National University, Acton, ACT, 2601, Australia; 4 Australian General Practice Network, Manuka, ACT, Australia; 5 National Centre for Primary Care Research Development Centre, Manchester University, Manchester, UK

**Keywords:** General practice, Private practice nursing, Physicians office, Spatial analysis, Skill mix

## Abstract

**Background:**

Changes to the workforce and organisation of general practice are occurring rapidly in response to the Australian health care reform agenda, and the changing nature of the medical profession. In particular, the last five years has seen the rapid introduction and expansion of a nursing workforce in Australian general practices. This potentially creates pressures on current infrastructure in general practice.

**Method:**

This study used a mixed methods, ‘rapid appraisal’ approach involving observation, photographs, and interviews.

**Results:**

Nurses utilise space differently to GPs, and this is part of the diversity they bring to the general practice environment. At the same time their roles are partly shaped by the ways space is constructed in general practices.

**Conclusion:**

The fluidity of nursing roles in general practice suggests that nurses require a versatile space in which to maximize their role and contribution to the general practice team.

## Background

The last decade has seen a significant increase in the number of practice nurses in Australia, doubling between the years 2003 and 2007 to 7,824. At the same time the number of practices employing nurses also doubled, with 60% of general practices now employing at least one nurse [1,2]. Australian general practices are usually small businesses, geographically separate and with considerable structural diversity [3]. Practice owners are usually GP’s, and nurses tend to be salaried employees. As a result practices need to fund nurse employment, along with other organisational costs, out of business cash flow.

The growth in general practice nursing has been facilitated by a number of Medicare rebates for nurse activity, incentives for rural practices and those in areas of workforce depletion to hire nurses [4], support from Divisions of General Practice [5,6], and the federal governments’ Nursing in General Practice Program. The rationale for providing these incentives was that nurses would solve health workforce shortages [7,8] and improve the quality of healthcare [9]. This move in Australia is part of a world-wide understanding that nurses benefit general practice, and that governments need to encourage them [10].

At the same time the general practice landscape is changing in response to professional evolution and health care reform. The doctor workforce is becoming increasingly feminised [11] and teamwork is widely promulgated as the way of the future [12,13]. Changes to average practice size and staffing configurations over the last decade have meant that pressure on general practice infrastructure has been intense [14], particularly in rural and outer urban areas where the bulk of nursing incentive payments have been directed [15].

One study identified concerns regarding the demands nurse are making on space requirements in general practices [16], where nurses are having to compete with doctors (often the owners of the practice), students, allied health and other staff. The aim of this study was to examine the effects of incorporating a large, new workforce cohort with a differently oriented professional culture to that of both general practitioners and past practice nurses into the spatial infrastructure of general practice.

## Method

This study undertook rapid appraisal of 25 general practices, spread throughout the two states of Victoria and New South Wales. 9 of the practices were from large metropolitan areas, 4 from regional cities, 11 rural towns and 1 remote practice. Rapid appraisal studies use a range of methods that share two features: they are field-based, and they are designed to be collected in a concentrated period of time. This makes them ideal for use in small, busy organizations [17]. The study was conducted between September 2005 and March 2006. Our study was approved by the human research ethics committees of the Australian National University and the Royal Australian College of General Practitioners.

Practices were identified by local divisions of general practice [6] in a purposive manner to identify practices expanding the nurse role. Informed consent from participants was obtained. During a day-long visit to each practice by a researcher, the following data were collected: interviews with nurses (n = 36), doctors (n = 24), and managers (n = 22); photographs of the general practice; two hours of structured observation of practice nurse activity (51 hours of observation of 34 nurses); practice floorplans and a situational analysis. Interviews were semi-structured, and addressed (for nurses) work history, types of work done in general practice, contribution to safety and quality, and their experiences within the workplace, and (for doctors and practice managers) the experiences of the general practice with nurses, roles of nurses and contribution to safety and quality. All interviews were audiotaped and transcribed. The rapid appraisal tool was piloted in two practices with each researcher and an independent observer; concordance rates for the observational component were 94% and 96%.

Further details of the sample, data collection and analytical methods have been presented elsewhere [18]. The sampling frame for this project was designed to include variation between practices in location, regional demographics, rural or urban orientation, size, business structure, GP & nurse workforce size, and types of nursing role.

Inter-case and intra-case analyses for each practice were performed by a multidisciplinary team (sociologist, nurses, GPs, policy analyst). All data, including photographs and floorplans, were coded into a database using NVivo qualitative data analysis software, version 7 (QSR International, Melbourne, Vic), enabling triangulated data interpretation. Whilst the research team could not be blinded to participants, anonymity has been protected by not reporting any identifying information as to practice whereabouts. All practices have consented to the publication of photos.

The research team probed for emergent themes, using the constant comparison method [19], and cross-checked with practice data. Primary themes included structural elements (health care policy, environment, gender, nursing culture); practice-level elements (interprofessional relationships, time-use patterns, space); and individual factors.

We then undertook a specific analysis of the influence of space, using the photos as referents, and correlating this with data from other elements of the study. The analysis of general practice spatial arrangements in this study is based on an interpretation of fixed images or fixed situations. According to Ball & Smith [20], fixed images (in this case, photographs and floorplans) can be interpreted according to their content (the elements of the photo), the referent (what the photograph is of), or the context (how the referent is used). By examining still shots of referents, we took the ontological position that the content exists not purely of itself, but that it carries with it its social context and can be interpreted. The images are therefore “lived visual data” [21], representations of three dimensional spaces that humans inhabit – in this case as a working environment.

## Results

The floor plans were classified first into different zones that could be used to analyse and interpret photos of different practice spaces:

Public – unrestricted areas (free access to all). Examples were the patient entry areas, including waiting rooms, and public access toilets.

Public – restricted areas (access restricted to control by staff). Examples included corridors behind consulting rooms.

Staff-only areas. Examples included reception and administration areas, tearooms and storage areas.

Clinical/Patient interaction areas. Examples included consulting and treatment rooms.

**Figure 1:**
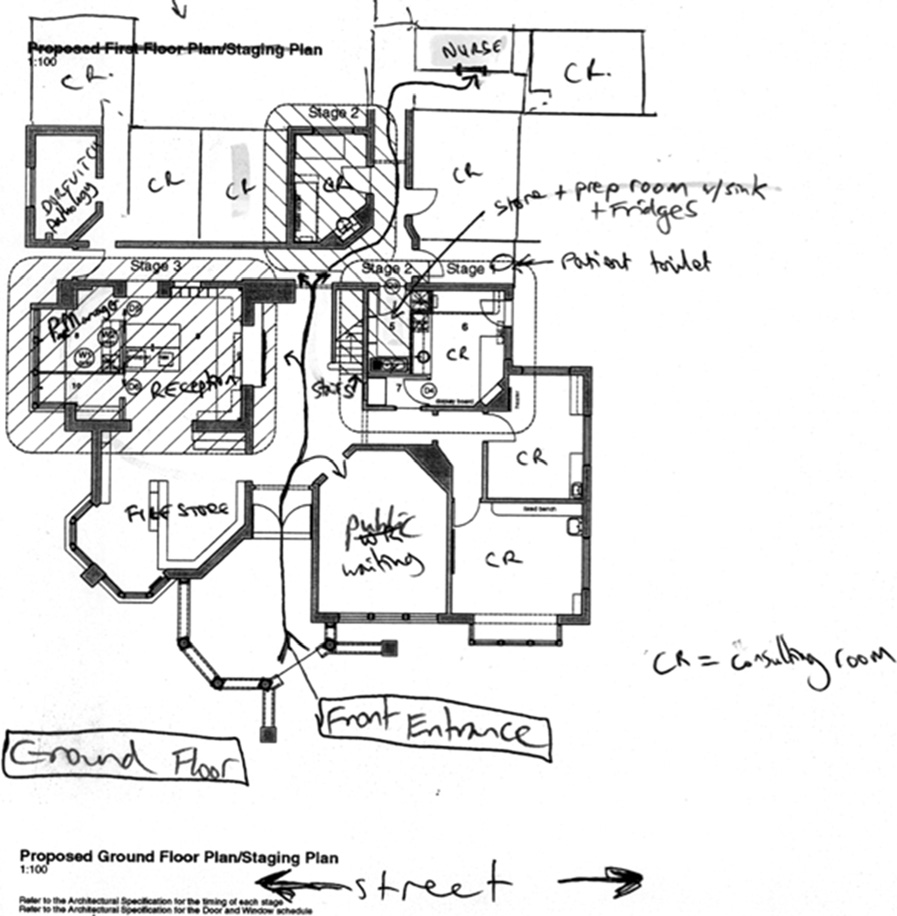
Floorplan, modified building.

It was quickly clear that practices in the study sample fell into two categories: practices that had modified an older existing building (often a house) to accommodate a medical clinic; and practices that had built (or extended) into a purpose designed clinic Figure 1.

**Figure 2:**
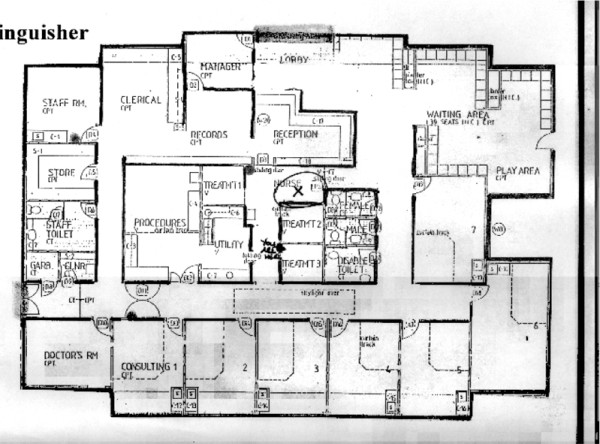
Purpose built practice.

Contrasting the floor plans in Figures 1 and 2, the differences between the two categories are highlighted. The first (Figure 1) is a long established practice, in a converted federation style house, in the inner suburbs of a metropolitan area. This practice has one nurse, and about 12 GPs. The entrance way is determined by the established front door of the house. Extensive internal work has been done to create consulting rooms, and the overall appearance is that of a ‘rabbit warren’. There is little clear delineation of the public unrestricted area, and public restricted areas. The nurse in this clinic has the smallest consulting room, and no specified treatment room. The ability for the nurse to carry out significant functions within the practice is limited by the practice design.

The practice in Figure 2 however, is purpose built. This is manifest in the clear identification of the four zones. Design consideration has been given to the working needs of the staff, and the nurse is given a central position. This structure was not unusual for these type of surgeries: waiting area to the front, consulting rooms around the edge, and a central island that contains administrative and nurse/clinical areas. In this case the nurse has her own administrative area, as well as having access to, and responsibility for, several spaces.

### How nurses use space: looking at the built environment

Within the surgery, nurses have a wide-ranging physical workspace, often wider than the doctors or reception staff. These areas reflect their combination of clinical and non-clinical roles. All but one practice had a treatment room as a centre of nurse clinical activity, whilst several also had dedicated consulting or clinical rooms. Many had an arrangement for time-sharing of consulting rooms when not in use by a doctor. While they clearly focused on treatment and consulting rooms, nurses also identified cupboards and stockrooms as key work areas, demonstrating a prioritisation of workplaces which were not “premium space” in the general practice.

**Figure 3:**
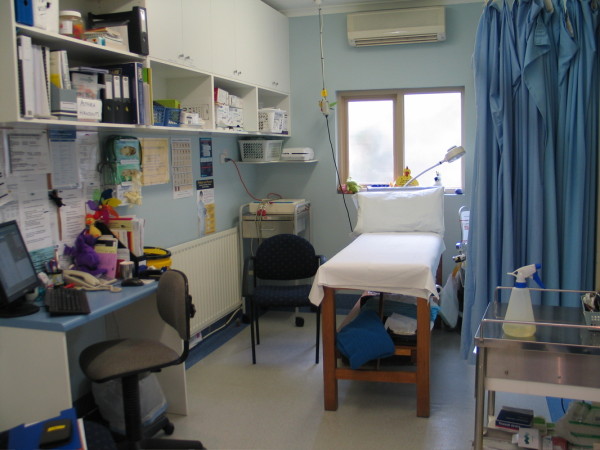
Treatment room.

The photograph in Figure 3 presents the most common workspace for nurses – the treatment room. It is in a four consulting room practice, and occupies a central or ‘hub’ position. Treatment rooms are often in a position that makes them a hub of activity within the practice, either amongst the consulting rooms or between the rooms and the reception area. The curtain seen in the right of the picture was the only form of visual privacy, and consequently conferred no aural privacy. This nurse works in a public (but controlled) environment. Observations revealed that other staff had little compunction in intruding in this space, quite distinct from their attitude to the consultation room. Examining the elements of the photos reveals much that is ‘intrinsically active’ - or designed to be used. These are occupational objects in the typology proposed by Riggins [21]. The predominance of clinical equipment and the organisation of the room indicate that this is a place where things are “done” to patients. There is very little personalisation of this workspace with esteem or social objects - objects conveying status or personality. The central object of this, and all treatment room photos, is the trolley or bed – reinforcing the centrality of the bed to nurse activity in this environment. The focus of this clinical workspace is on the patient – and the procedure to be undertaken - the nurse is secondary. This is in contrast to many medical consulting rooms that display personal artifacts important to the individual practitioner.

**Figure 4:**
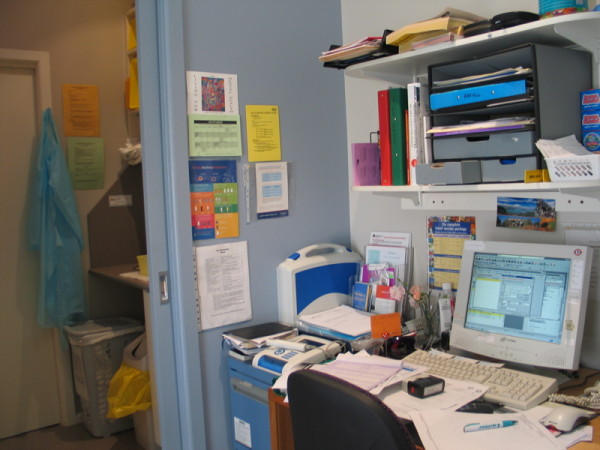
Nurse work station.

By contrast, the nurse desk in Figure 4 is not in a primarily clinical area, but in a shared administration area, adjacent to storage rooms. Here, there is much more ‘intrinsically passive’ material, with social objects found in the form of the jellybean jar and the pot plant. A common feature of all these workspaces is the presence of posters/handouts stuck on the wall. You can see here several sheets of information on the wall, information that the nurse feels she needs rapid access to. This is mirrored in the first photo, with the space above the desk covered with information. In the first photo, note also on the other side of the gloves is more generic information that is more available to the patient and may be less frequently referred to.

**Figure 5:**
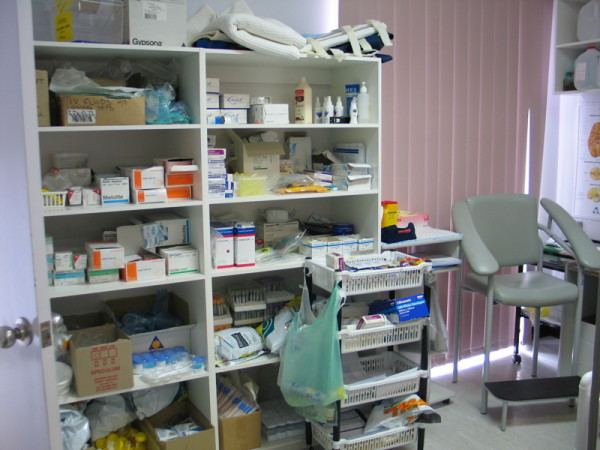
Clinical storage area.

A third category of nurse workspace identified in this study is storage or utility areas. These may be integrated into existing spaces or occupy separate areas/cupboards, but are typically like the one shown in Figure 5 – containing almost exclusively active, occupational objects. Their frequent presence in the photos is a reflection of their prominence in nurses’ work practices, both in ensuring stock is kept up to date, and in managing patients in a clinical environment. Nurses frequently reference this aspect of their work and see it as an underpinning component of their contribution to the practice team.

The overall impression is that nurses enact their work in three spatial domains: clinical interactions and activity primarily within patient-centred spaces; administrative work in an ‘office’ setting, and equipment, stock management and support activities conducted within the ‘backstage’ areas of a practice. Correlating this information with the observational and interview data reinforces the fluidity of the nurse space, with nurses cycling through multiple tasks in a given time frame, and deploying a series of operating roles as they adapt to the practice environment.

The six primary operating roles of general practice nurses identified in this study have been described elsewhere [18], but include: patient care; organisation; problem solving; quality control; education and connectivity. Nurses move consistently through practice space as they cycle between these operating roles. The physical characteristics of the practice space influence these roles through the positioning of nurse spaces, their accessibility, and the independence or shared nature of space utilization. In particular, these elements affect the interconnectedness of nurse work with that of others, and the strength and utility of their connectivity role. This role is an important capacity factor in many practices and an enabler of extended relationships with patients and the broader community.

**Figure 6:**
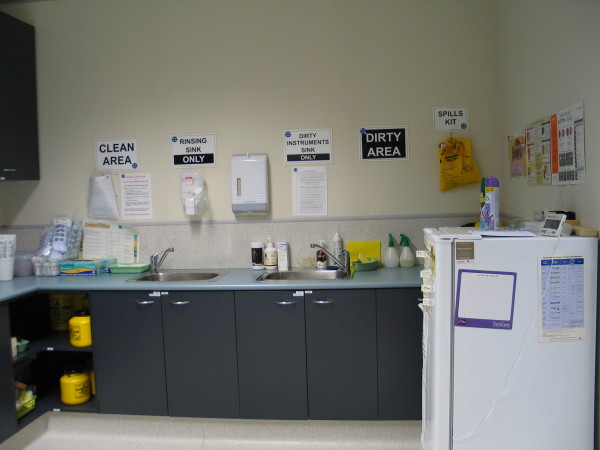
Treatment room.

Nurses are frequent sharers of space – either with other nurses or with colleagues from other disciplines including GPs. In this situation they commonly appropriate elements of the space to be used in a certain way and act as monitors of the behaviour of other space users. This is illustrated by the signage in Figure 6 that acts as both a communication and quality control mechanism.

### “A free consulting room is like a present”: talking about space

A prominent theme in the interview and observation data was the lack of space available in general practice to perform the tasks required of the practice team.

Nurse: “[We] don’t need more staff. More than anything we need more space. And -”

Interviewer: “So another treatment room ?”

Nurse: “No, we need - well, a bigger treatment room would have been good but we also need more consulting rooms. I think we could have done with at least one or two more consulting rooms” *[PN, practice 3]*

This space shortage applied to many activities of the practice as a whole, as well as to nurses specifically*.*

"“Room, space is…that would be the most difficult thing here, lack of space it just makes everything difficult. I don’t know if you noticed this morning I went and asked if there was any free consulting rooms, you know if there is a free consulting room it is sort of like a present” *[PN, practice 2]*"

The influence that this had on nurse practice was generally acknowledged, and several of the interviewees noted that, in the scheme of practice allocations, doctors (as greater income generating units) would always have priority over nurses. This was particularly evident in practices based on a modified house design, such as in Figure 1. Nurses in those practices often described them as ‘rabbit warrens” or “cramped and cosy”. Those in surgeries that had been renovated or purpose built had a more positive view of the space allocation. In the following account, a nurse in a rural area describes the section of the clinic where she works, which has been purpose-built with central cubicles and a nursing desk.

Nurse: “[W]e have a designated nurses area where we have a main desk with our computer system where we do all of our own filling and obviously all the encounters and things for every patient is on the computer.…”

Interviewer: “So that’s almost like a front desk, isn’t it?”

Nurse: “It is, yes. So patients come to us to report, which they don’t really need to but they all like to report to certain doctors…And then from there we have the sterilising area which has also got where we keep all of our medications from the drug reps and things like that, and stock in that area, which goes out to the theatre, which joins onto the theatre which also has a lot of our stock in there.” *[PN, practice 13]*

Nurses were often involved in the designs of renovations and new surgeries, and this was clearly associated with higher satisfaction with the space allocation. In the following account, a nurse in a large multi-doctor and multi-nurse practice describes a process where the nurses limited their accessibility to patients while maximizing the contiguousness of working patterns with other nurses.

"“The initial plan by the architect just wasn’t - wasn’t very good. Well, we didn’t think it was. And we had a nurses meeting one night because we kept saying to them no, it’s not good enough, we’ve got to walk around too much. And in the original planning they actually had the nurses area directly linked to the patient waiting area and so we would have been walking in and out the patient waiting area, you know, like - and you couldn’t get to the tea room without people sitting and watching you going [and] having cuppas, [that] sort of thing. So we had a meeting one night at one of the nurse’s homes and we actually sat down with the plan and redrew our nursing area. And what we’ve got basically is what we drew up.” *[PN, practice 6]*"

We have already noted the diverse spaces in which general practice nurses work, characterised by a much more mobile, accessible, open work orientation, especially when compared to the closed, consulting room settings favoured by doctors. Such an open environment was a feature of both the older style surgeries, and the purpose built ones. Nurses are clearly comfortable with, and in fact favour, such an open environment. They saw it not just as a task related feature, but as an expression of their accessibility and the candid or relaxed relationship that perceive they have with patients.

"“[W]e don’t put up barriers for people. We don’t make it difficult for them. We don’t make them feel like ooh, I’m overstepping the mark here, I shouldn’t be here or - yeah, make it very warm. It’s like walking into a home, this place, yeah, and I think that makes a big difference.” *[PN, Practice 3]*"

**Figure 7:**
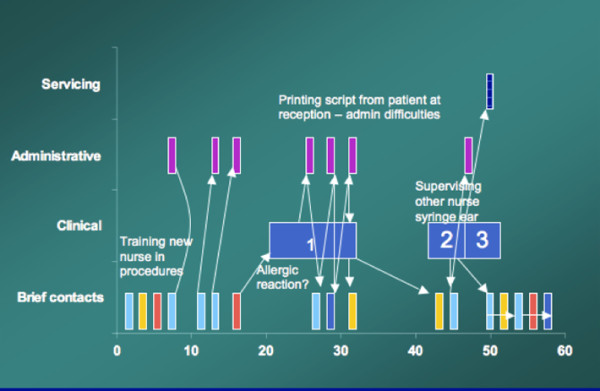
Space and the rapid cycling of nurse tasks.

The example in Figure 7 shows how rapid cycling of tasks is enabled by working in an open environment. The space requirement of this nurse took her from treatment room through shared and administrative spaces, but importantly her availability in a shared space allows the connectivity element to occur. Doctors are locked away in consulting room, requiring a knock and permission before entering, Nurses, by virtue of the combination of their role and accessibility in the practice space, are available for more unstructured staff and patient contact.

## Discussion

Nurses require a larger floor space than doctors, because of the fluid and variable nature of nursing work, At the same time, they are more mobile and spend less time in a single location. Nurses work across all space zones in a practice, and often cycle through these repetitively in such a way that they seem to have an ‘access all areas’ pass. This reflects the rapid cycling of tasks that they have been observed to undertake, and the interconnectedness of their interactions with other members of the practice team^27^. Nurses also perceive their workspace in a broad way, including storage, utility and medical supply areas.

Practices tend to locate nurses in a treatment room or other central, procedural or transitional space. In many cases this is the result of space pressures in a practice where the availability of space is limited by design, geography or cost. Most urban, and many rural, general practice nurses work within modified houses, and often have limited access to consulting spaces. The transitional or shared nature of the spaces they routinely occupy means that privacy may be compromised. This is further compounded in cases where space is structured to facilitate easy access and mobility such as curtains and open areas, and where the space is freely entered and utilized by others, such as doctors. This limits their capacity to expand their patient carer role through individual consultations in a way that is typical of general practice, but enables and fosters their connectivity role.

Nurses demonstrate high levels of adaptability to the spaces they are allocated, and are able to adapt and modify their workspaces to fit the needs of their workflow. Most nurse workspaces in this sample, however constrained, had been organized to achieve high levels of functionality and often served as verbal and non-verbal communication hubs. This may reflect the collective orientation of nurses [22], stemming from their professional culture as a numerate, interchangeable workforce – especially in hospital settings [23].

However there are spatial configurations which foster greater communication and teamwork. Iedema et al [24]. have suggested that meaningful teamwork is conducted in transitional or liminal spaces such as the corridor, where “the interstices among clinical knowledge, processes, problems and purposes are dynamically negotiated and worked out”. In this study we found that designs that isolated or removed nurses from a central location tended to reduce the incidental involvement of nurses in practice life, and limited their connectivity.

In contrast to their ownership of workspaces, nurses have less control over the floorplan and structural layout of practices. Decisions about space, which are often also decisions about income, expenditure or resource utilization, are generally made by practice owners, although they may be made in consultation with the existing nurses at the time of planning. Nurses, within current funding structures, are less able to generate revenue that GPs, and as such may be seen to represent a less efficient use of space, despite their need for wide ranging and flexible work spaces.

As practice teams and general practice nursing roles evolve, surgery design (and modifications to existing surgeries) will need to take into account the many factors that we have considered here. The workspaces that nurses inhabit have arisen not just out of a historical role, but developed in response to their multiple roles within the practice. The previously highlighted connectivity and trouble shooting roles require accessibility, and accessibility come from nurses inhabiting those public, but controlled environments. What nurses require is space flexibility in accordance with their role flexibility – private space when the work demands, public space at other times.

### Limitations

This study does not purport to be a detailed and representative analysis of all Australian general practice. By identifying practices at the developing edge we hoped to identify information to inform change across the rest of the sector. The strength of the method is in the multiple data sources, yet perforce each source has its own limitations, be it the perceptions of participants or the practice dynamics on the day, which may be influenced by the time of year, etc.

The study was conducted some years ago, which raises the question of its current validity – however the study was done with practices who were effectively ‘early adopters’ in a process that continues to evolve through the Australian health care system. Similarly, as this is a study of change in general practice, we do not feel the results will date.

## Conclusion

Nurses in general practice have fluid and dynamic roles that are enacted across three main spatial domains: the consultation space, the administrative space and the ‘backstage’ practice environment. The fluidity of these roles and the mobility of nurses’ transition through surgery space are an important part of their contribution to general practice organisational life, and a result of the impact of their professional culture on the GP environment. Infrastructure constraints (either lack of space or the money to enhance it) are significant issues for general practices in the current reform environment where the nature and structure of practice teams is under pressure to change – and mainly grow. The fact that nurses are versatile personnel who require versatile workspace may be a key consideration for policy makers who want to facilitate the development of teamwork in general practice.

## Competing interests

The authors have no competing interests.

## Authors’ contributions

All authors were involved in design and analysis of the study. CMP wrote the first draft of the paper and all authors contributed to its development. All authors read and approved the final manuscript.

## Pre-publication history

The pre-publication history for this paper can be accessed here:

http://www.biomedcentral.com/1472-6955/11/13/prepub
